# Distributed Acoustic Sensing Using Dark Fiber for Near-Surface Characterization and Broadband Seismic Event Detection

**DOI:** 10.1038/s41598-018-36675-8

**Published:** 2019-02-04

**Authors:** Jonathan B. Ajo-Franklin, Shan Dou, Nathaniel J. Lindsey, Inder Monga, Chris Tracy, Michelle Robertson, Veronica Rodriguez Tribaldos, Craig Ulrich, Barry Freifeld, Thomas Daley, Xiaoye Li

**Affiliations:** 1 Lawrence Berkeley National Laboratory, Energy Geoscience Division, California, USA; 2 University of California, Berkeley, Earth and Planetary Sciences Department, California, USA; 3 Lawrence Berkeley National Laboratory, Energy Sciences Network, California, USA; 4 Lawrence Berkeley National Laboratory, Computational Research Division, California, USA

## Abstract

We present one of the first case studies demonstrating the use of distributed acoustic sensing deployed on regional unlit fiber-optic telecommunication infrastructure (dark fiber) for broadband seismic monitoring of both near-surface soil properties and earthquake seismology. We recorded 7 months of passive seismic data on a 27 km section of dark fiber stretching from West Sacramento, CA to Woodland, CA, densely sampled at 2 m spacing. This dataset was processed to extract surface wave velocity information using ambient noise interferometry techniques; the resulting *V*_*S*_ profiles were used to map both shallow structural profiles and groundwater depth, thus demonstrating that basin-scale variations in hydrological state could be resolved using this technique. The same array was utilized for detection of regional and teleseismic earthquakes and evaluated for long period response using records from the M8.1 Chiapas, Mexico 2017, Sep 8th event. The combination of these two sets of observations conclusively demonstrates that regionally extensive fiber-optic networks can effectively be utilized for a host of geoscience observation tasks at a combination of scale and resolution previously inaccessible.

## Introduction

Hydrogeologic and seismological data collection are two domains for which the absence of high spatio-temporal resolution data is particularly acute, with significant impacts on our ability to characterize near-surface soil properties, groundwater systems, and seismic events. Even relatively basic subsurface hydrological parameters such as water table depths in surficial aquifers suffer from severe undersampling in both space and time. While heavily monitored basins often have a multitude of wells providing subsurface access, they are neither uniformly distributed nor frequently monitored resulting in heterogeneous datasets requiring manual quality control, curation, and analysis. The few basin-wide hydrogeological data sources, typically based on satellite remote sensing technologies, provide only surficial property estimates like soil moisture^1,2^, integrated strain response (e.g. InSAR^3^), or low-resolution volumetric datasets (e.g. GRACE^4,5^) which require assimilation with point measurements to provide finely resolved operational parameters^6^. Remote sensing measurements often also suffer from temporal undersampling due to satellite pass frequency. More recently, seismic ambient noise interferometry^7^ has been leveraged to provide broader information on ground water storage; unfortunately limited permanent seismic networks present a challenge for these approaches.

Likewise, seismological data collected using existing permanent networks often have spatial regions which suffer from significant spatial undersampling, particularly in areas distant from major plate boundaries, resulting in challenges when attempting to detect and locate small natural and induced events. The case of small magnitude induced events is particularly problematic since the basins where oil and gas production, wastewater injection, and carbon dioxide sequestration occur are often distant from historically seismogenic faults and the associated permanent seismic networks. Network sparsity increases the minimum event size for detection, results in statistical biases in the catalog, and greatly increases depth uncertainty for local events. Recent studies focusing on seismic catalog completeness in California have determined that even M2 events cannot be detected in the majority of the Sacramento and San Joaquin Basins using the existing network stations^8^.

These spatio-temporal undersampling problems, for both hydrological and seismological measurements, can be remedied by re-purposing ubiquitous sensing platforms already deployed at scale. A recent example of such an approach is the utilization of smartphone accelerometers to measure strong ground motion as part of earthquake early warning applications as shown in previous studies^9,10^; other examples include using social media proxies as sensors^11^ or MEMS accelerometers in pervasive stationary devices such as personal computers^12^. Broader efforts to leverage networking and sensor technologies related to the Internet-of-Things (IoT) for seismology are developing but still in their infancy^13^.

An alternative approach is to exploit components of the built environment to serve as distributed sensor networks. In this case we couple the use of unlit subsurface fiber-optic cables, commonly referred to as “dark fiber” since they are not utilized for data transmission, and distributed acoustic sensing (DAS) to provide such a spatially extensive sensing platform. The vast majority of fiber-optic cables in the earth’s near-surface were installed exclusively for the purpose of telecommunications. Due to high cost of fiber-optic installation, typical commercial practice is to deploy significantly more capacity, as measured by fiber count, than required; this practice, combined with advances in bandwidth available per fiber, have yielded a surplus of available fibers that remain unused. The US footprint of such unused fiber networks is massive with tens of thousands of linear kilometers of long distance fiber-optic cables available for lease or purchase in the current environment. One notable aspect of such dark fiber network components is that they tend to utilize existing “right-of-way” corridors along roads and rail connections^14^, environments rich in ambient seismic noise. Given the ubiquitous nature of installed telecom fibers, few studies have explored use of this resource for sensing applications. An early experiment explored the use of Brillouin Optical Time Domain Analysis (BOTDA) to monitor temperature over previously installed telecom fiber^15^; however, these studies were conducted primarily to provide network integrity information rather than for environmental sensing. In a seismological context, several recent studies^16–18^ have demonstrated the benefits of leveraging urban telecom infrastructure at a small scale. Most recently, a study in southwest Iceland^19^ provided an excellent example of utilizing telecom fiber for detecting local earthquakes and measuring co-seismic strain measurement over a short transect.

Distributed Acoustic Sensing (DAS) is a recently developed technique which utilizes coherent optical time-domain reflectometry to accurately measure the phase and amplitude of vibrations along an optical fiber^20–22^. The technique exploits changes in Rayleigh scattering induced by extensional strain; these measurements have now been quantitatively compared to point seismic recordings at both intermediate^23^ and low frequencies^24^ and utilized for a host of tasks including vertical seismic profiling^20,21,25^, near-surface soil property estimation^26–30^, surface refraction tomography^31^ and earthquake seismology^16–19,32^. DAS has created a recent paradigm shift in applied geophysics by enabling seismic measurements at a combination of high frequency (kHz range), large distances (tens of km), and fine spatial sampling (as small as 1 m), a combination previously unavailable with conventional sensors at moderate costs. We should note that DAS is distinct from long-range optical interferometry approaches which provide even greater measurement distances but sacrifice spatial localization; this class of techniques was recently demonstrated^33^ as an approach for seismic detection utilizing trans-oceanic cables. While prior studies have convincingly demonstrated the value of dense networks for seismic imaging as well as a range of other purposes^34^, the high costs associated with massive nodal deployments over long time periods has precluded their use in many contexts.

In this study, we demonstrate the application of DAS utilizing dark fiber for measurement of seismic wavefields at the sub-basin scale with an extremely fine spatial sampling (2 m) over long time periods; the resulting ultra-dense dataset is utilized for both hydrogeological/near-surface characterization, using ambient noise interferometry, and the detection of seismic events, both regional and global. This combination is perhaps a new frontier which leverages investment in built infrastructure to greatly extend the reach and sampling of existing permanent monitoring networks.

## Seismic Monitoring with Dark Fiber Networks

**Figure 1:**
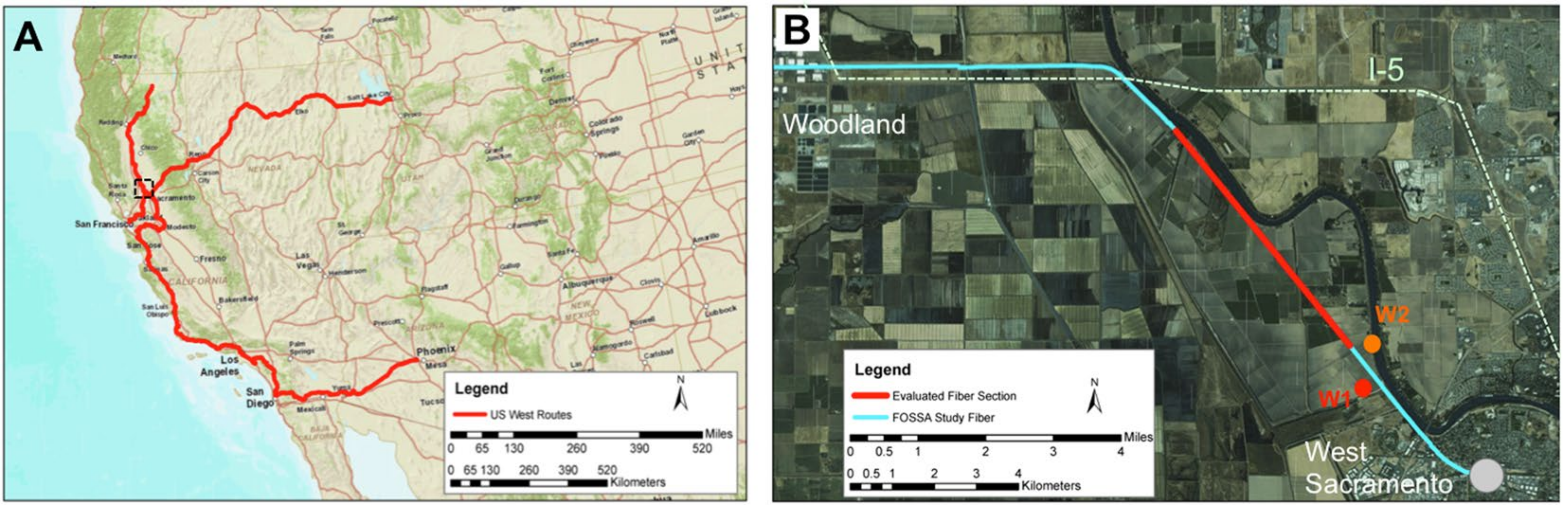
Map of a section of the ESNet Dark Fiber Testbed (https://www.es.net/network-r-and-d/experimental-network-testbeds/100g-sdn-testbed/terms-and-conditions/). (**A**) The regional network within CA and western NV; zone of panel (B) shown in black dashed box. (**B**) The subsection of the network used in this study. The red segment in (**B**) is the area of focus for ambient noise analysis; W1 and W2 are reference wells for water table and soil horizons, respectively. The study fiber (blue) is aproximately co-linear with an active rail line. Dashed green line labeled I-5 is Interstate 5, a major source of ambient noise beyond the rail corridor.

Our study utilized dark fiber components of ESnet’s Dark Fiber Testbed. ESnet, a US Department of Energy (DOE) user facility, provides high-performance unclassified network infrastructure to connect DOE research sites including high performance computing (HPC) facilities and data-intensive instrumentation e.g. x-ray, neutron, and nanoscience facilities. The Dark Fiber Testbed is a 20,920 km (13,000 mile) network of short and long haul telecommunication fiber designed for testing novel network communication equipment and protocols. The network consists of single mode telecommunication fibers of varying age and installation technologies and hence is an excellent proxy for existing commercial network components. This study is one of the first experiments that utilizes this massive network for sensing purposes. Figure 1A, depicts the long haul regional sections of the Dark Fiber Testbed in California, as well as the segment exploited for our test (1B), which runs from West Sacramento, CA to Woodland, CA.

## Field Deployment

The site of our study was a transect located in the Sacramento River flood plain, north and west of Sacramento, CA. The geology of the site consists largely of Quaternary sediments including a sequence of silts and clays underlain by fine sands. Prior regional studies^35^ have mapped the surface sediments as a mixture of poorly sorted Holocene alluvium near the Sacramento and finer-grained Holocene basin deposits deeper in the flood plain. Partially lithified sediments from the Tehama formation have been mapped from approximately 50 m to greater depths^36^. The segment of dark fiber we utilized for this study, shown in Fig. 1 panel B in blue, runs from West Sacramento CA to the small town of Woodland CA. As can be seen from the fiber network map, the recording profile extends from an urban environment into a section of farmland near the Sacramento River, crossing Interstate 5 before bending westward towards Woodland. For the length of the fiber route shown in Fig. 1B, installed cables utilize the right-of-way associated with a rail line and are roughly co-linear with the train tracks. The agricultural areas sampled by this profile are partially irrigated through a variety of methods and groundwater is actively extracted from both the shallow surficial aquifer as well as deeper sources.

The seismic dataset presented in this study was recorded between July 28th, 2017 and Jan. 18th, 2018. The DAS interrogation unit (IU; Silixa iDAS, Elstree, UK) was installed in a telecommunication Point-of-Presence (PoP) facility in West Sacramento. Hardware details on the installation are described in Methods Section 0.2. Ambient seismic noise was recorded using the DAS IU at 500 Hz sampling with a spatial sampling of 2 m; the gauge length was 10 m and fixed in hardware. Data was streamed continuously to large capacity (8 TB) USB-3 external hard drives that were exchanged on a weekly basis.

While the surface geometry of the dark fiber network was known before deployment, the mapping to linear fiber location was established by sequential impact tests at surface locations surveyed with high accuracy differential GPS. This is necessary due to the common practice of including spools of slack cable during telecom installation, an approach that makes the mapping of surface geometry to linear fiber location more complicated. Impact locations were observed on individual DAS gathers in terms of fiber distance, coordinated by GPS time; by establishing true DGPS coordinates for these locations, we were able to compensate for slack effects when mapping back to the previously surveyed deployment geometry. The resulting geometry likely has an uncertainty on the order of 5 m due to the interpolation process along the transect.

After the network geometry was established, DAS signal strength, amplitude, and periodicity were examined to evaluate noise characteristics along the array. Dominant noise features include several regional highways, diffuse urban noise, and energy from local railroad activity. Qualitatively, the highest quality data was observed on a straight section of fiber starting beyond West Sacramento and extending to the noise field of Interstate 5, shown as the highlighted red profile in Fig. 1B. Zones to the Southeast of this section suffered from non-optimal installation conditions (e.g. the fiber was attached to surface structures including a bridge) as well as incoherent noise in the urban transition zone around West Sacramento. Zones to the North and West suffered from both optical fading, insufficient return photons which decreased measured S/N, and broadside noise interference from Interstate 5. This illustrates the potential heterogeneity of signal quality across the existing telecom network.

## High Resolution Kilometer-Scale Near-Surface Imaging Using Ambient Noise

Prior studies have demonstrated the potential of utilizing ambient noise interferometry and classical sensors to detect fluctuation in groundwater state^7^. Ambient noise interferometry is an established group of seismological techniques that utilize environmental vibrations from either near-surface or subsurface sources to retrieve coherent seismic information, referred to as empirical Green’s functions^37–41^. More recently, several authors^26,28^ have demonstrated that such techniques could be utilized to transform infrastructure noise in the 2–30 Hz band (e.g. surface waves generated by cars, trucks, and trains) into accurate and stable 1-D estimates of shear wave velocity from 0–30 m depth using DAS. While prior studies have examined data acquired on a fit-for-purpose array, the processing strategy proposed can be easily adapted to dark fiber deployments.

**Figure 2:**
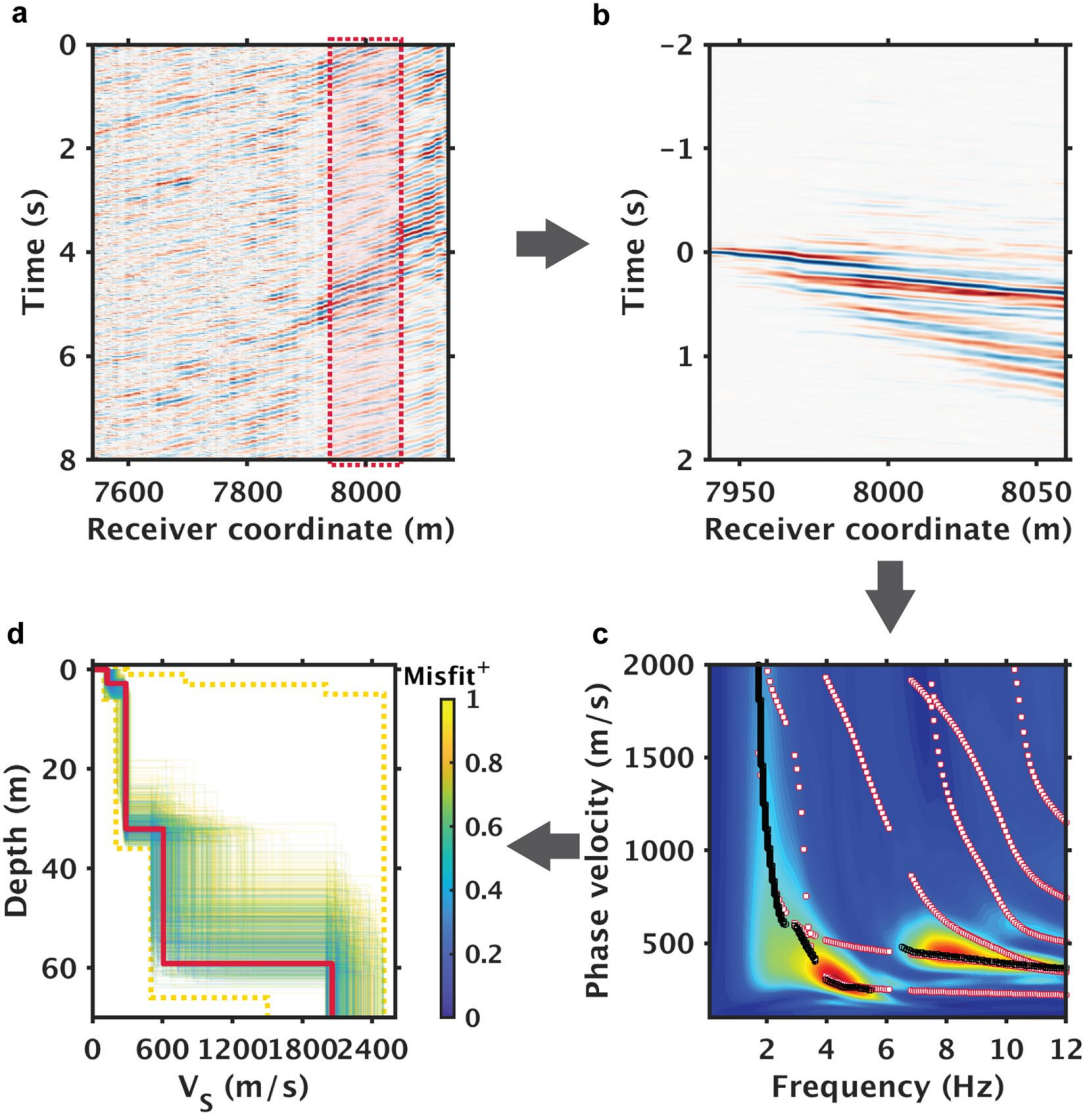
Illustration of data processing workflow for ambient noise interferometry. (**a**) Example of train noise shown via an 8 second time domain slice. The red box in (**a**) highlights subsection of the array used for (**b**) noise correlation gather, (**c**) dispersion analysis, and (**d**) inversion of the shear-wave velocity (*V*_*S*_) profiles. Black and white markers in (**c**) denote observed and model-predicted multimodal dispersion curves respectively. In (**d**), the yellow dashed lines denote upper and lower bounds of the parameter space used in Monte Carlo sampling; the bold red line marks the best-fit *V*_*S*_ profile; the yellow/blue lines denote the top 0.1% best-fitting *V*_*S*_ profiles (color coded by their corresponding inversion misfits); Misfit^†^ denotes normalized misfit values (min-max normalized by misfits of the top 0.1% *V*_*S*_ profiles).

The present study images near-surface shear wave structure using infrastructure noise generated by freight trains operating along a 6600 m subsection of the dark fiber array (bold red line in Fig. 1B). Similar to earlier work which exploited road noise, we use ambient noise interferometry to transform the raw noise records into virtual common-shot gathers; we then apply multichannel analysis of surface waves (MASW) analysis^42–45^ to infer shear-wave velocity (*V*_*S*_) profiles with a multimodal inversion strategy. Figure 2 is an illustration of this workflow (see Fig. S1 for more details). All examples use only 40 minutes of ambient noise data with the caveat that only noise from trains are utilized; a more detailed discussion of data selection is detailed in the Methodology section. After generation of dispersion images from virtual common-shot gathers, we auto-pick dispersion curves for high energy modes. These experimental curves are inverted using a previously developed Monte Carlo (MC) inversion strategy^46^ which utilizes a novel multimodal objective function^47^ which does not require mode numbering. Supplementary Fig. S2 shows a detailed example of solutions across the array and associated fits to experimental curves. Of the resulting solutions, the best-fitting model is selected for interpretation; however, the family of accepted models can be used to evaluate solution uncertainty as discussed in the Supplementary section and shown in Fig. S3.

**Figure 3:**
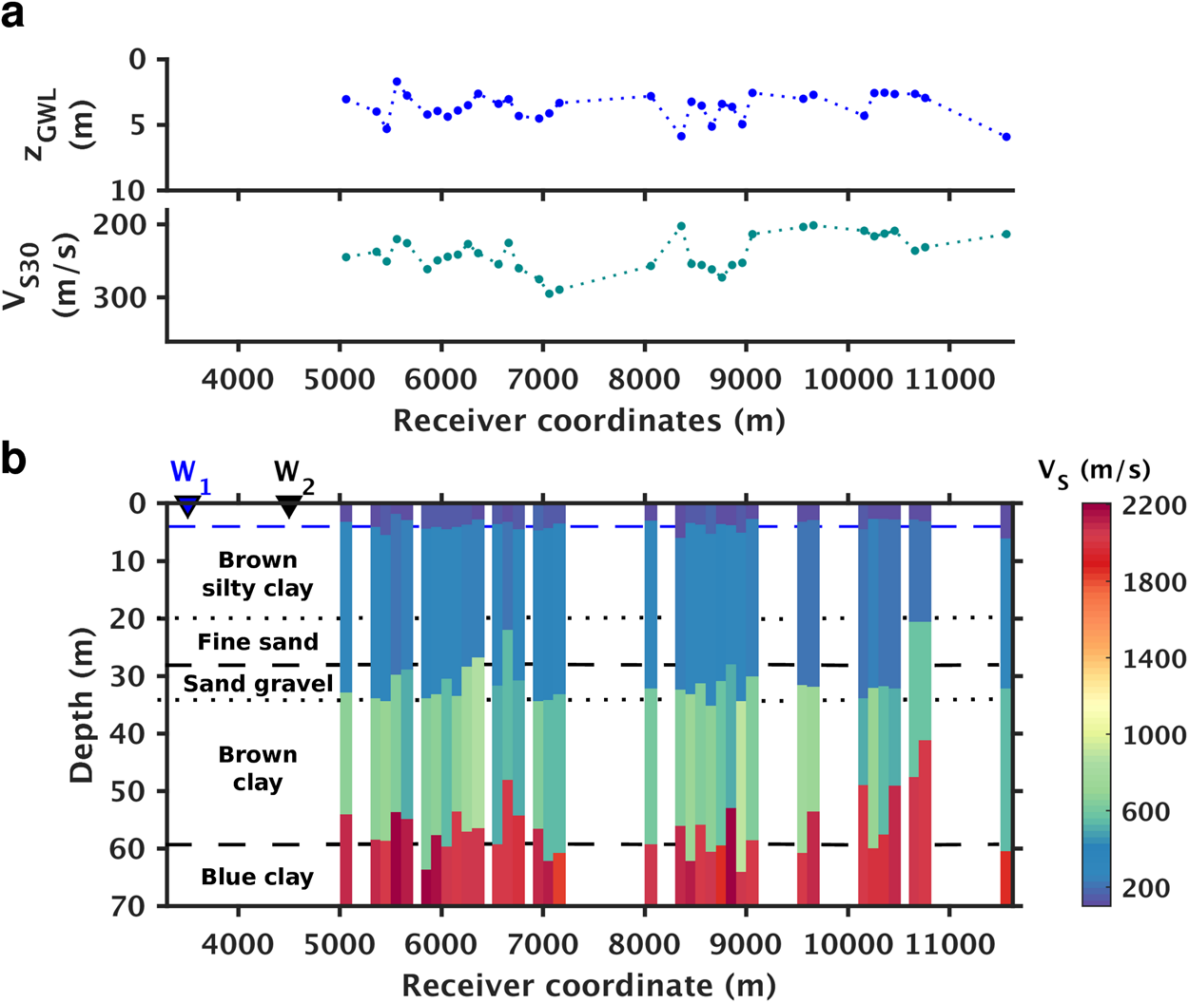
Shear-wave velocity (*V*_*s*_) inversion results and ground truth comparison. (**a**) Depths of groundwater levels (GWL) (upper) and *V*_*s*_30 estimates (lower) extracted from the surface wave inversion results. (**b**) Pseudocolor display of *V*_*s*_ profiles and comparisons against well data. In (**b**), W1 and W2 mark surface locations of the two reference wells shown in Fig. 1; the blue dashed line denotes the depth of the ground water level provided by water well W1; the black dashed lines correspond to lithological horizons obtained from geotechnical well W2.

The results of our processing flow are a series of *V*_*S*_ profiles, each representing 1-D approximations of the subsurface underneath each of the 120-meter-long fiber subsections (Fig. 3). This length was selected to provide sufficient array size for dispersion analysis yet some degree of lateral spatial resolution^42^. As is to be expected, the installation conditions of the dark fiber are not always ideal for *V*_*S*_ imaging, hence gaps are left in the pseudo 2-D section shown in Fig. 3b. Reasons for the gaps include a small section with localized strongly directional coherent noise, likely a pump in one case, as well as poorly-structured dispersion curves which could not be fit within reasonable tolerance. However, with 57% of the 6600-meter-long fiber transect providing useful data (inverted sections in Fig. 3b), our lateral coverage of 3760 meters warrants its place as one of the longer high-resolution MASW profiles obtained using ambient noise alone. For all offsets shown, the forward modeling based on the inversion results effectively predicts the picked dispersion curves as can be seen in Fig. 2 as well as Fig. S2 for the fundamental mode and 1st overtone which can be reliably observed. We should note that the S-wave velocity of the lowest interface (underlying half space) is poorly constrained due to the limited offset, minimal recovered energy at low frequencies, and biases in the autopicker under these conditions; autopicker behavior within our present implementation tends to bias this velocity high without manual intervention. Zones above 40 m are considerably better constrained as discussed in the Supplementary section 2.

Comparisons to our limited ground truth database reveal that the interfaces of the *V*_*S*_ profiles match reasonably with the groundwater level and deeper lithological horizons, which confirms the validity of the inversion results. The lithological horizons consist of transitions from near-surface silty clay units to deeper sand/gravel units and underlying clay-rich horizons, as inferred from drilling logs from an unmonitored local well (W2) as shown in Fig. 3b. The depth to the surficial aquifer is estimated from the thickness of the top low velocity layer recovered from the surface wave inversion; in Fig. 3a, these estimates are compared to the water table depths measured in well W1, located slightly to the southeast of the measurement section. Details of well W1 are included in the Supplementary Information. More detailed analysis of a sub-array in the central section of the profile (see Fig. S3) show that the depth uncertainty for the top interface at that location is approximately 4.62 ± 0.8 *m*, hence the measured water table matches within error.

Also shown in Fig. 3a is an estimate of *V*_*S*30_, the travel-time average of *V*_*s*_ over the top 30 meters, calculated directly from the surface wave inversion results. *V*_*S*30_ is a widely used indicator of seismic site conditions and is readily obtainable from surface wave inversion^48^; both the values and the lateral variations of *V*_*S*_30 are useful data products that the dark fiber array can provide and can be compared to local estimates made as part of geotechnical studies.

To evaluate the utility of the DAS ambient noise inversion results for hydrogeological monitoring, we compare the transition in *V*_*s*_ identified as the water table to direct point measurements in the one well with sufficient temporal sampling information located on our transect. The well (W1) is located at the southeastern end of the high S/N section of dark fiber near the intersection of County Road 126 and the Old River Road, shown as a red dot on Fig. 1B. Water level in this well varies by as much as 7.6 m (25 ft) over annual hydrologic cycles and is impacted by Sacramento River levels, agricultural irrigation and surficial aquifer pumping. As can be seen in Fig. 3a, the depth of the first *V*_*s*_ increase (3.8–4.6 m below the surface) correlates to the measured surficial water table depth. We should note that an increase in *V*_*s*_ at the water table is not predicted by classical rock physics models which assume that shear modulus is not sensitive to saturation; despite this, past field studies^26,49^ have provided detailed observations of such sensitivity, presumably due to the impact of moisture on soil cohesion for dry surficial materials.

## Time-Lapse Monitoring of Groundwater Level

As mentioned previously, the Sacramento Basin is a dynamic hydrologic environment with multiple productive aquifers, active groundwater production, irrigation, and river interactions. Besides static profiles quantifying *V*_*s*_, the dark fiber array also allows for time-lapse monitoring of the subsurface, enabling measurement of hydrologic transients in the near-surface. Traditionally, because sensors are rarely dense enough for imaging, time-lapse monitoring results are typically presented as an apparent-velocity perturbation along the path connecting two sensors, without identifying where on this path the changes are occurring. This level of detail is insufficient for proactive management of groundwater resources, given that both vertical and lateral changes of the groundwater level could affect the overall sustainability of the aquifer. With the dark fiber profile enabling time-lapse imaging of the near surface, changes in groundwater levels could be resolved with spatial-temporal resolutions pertinent to groundwater management.

**Figure 4:**
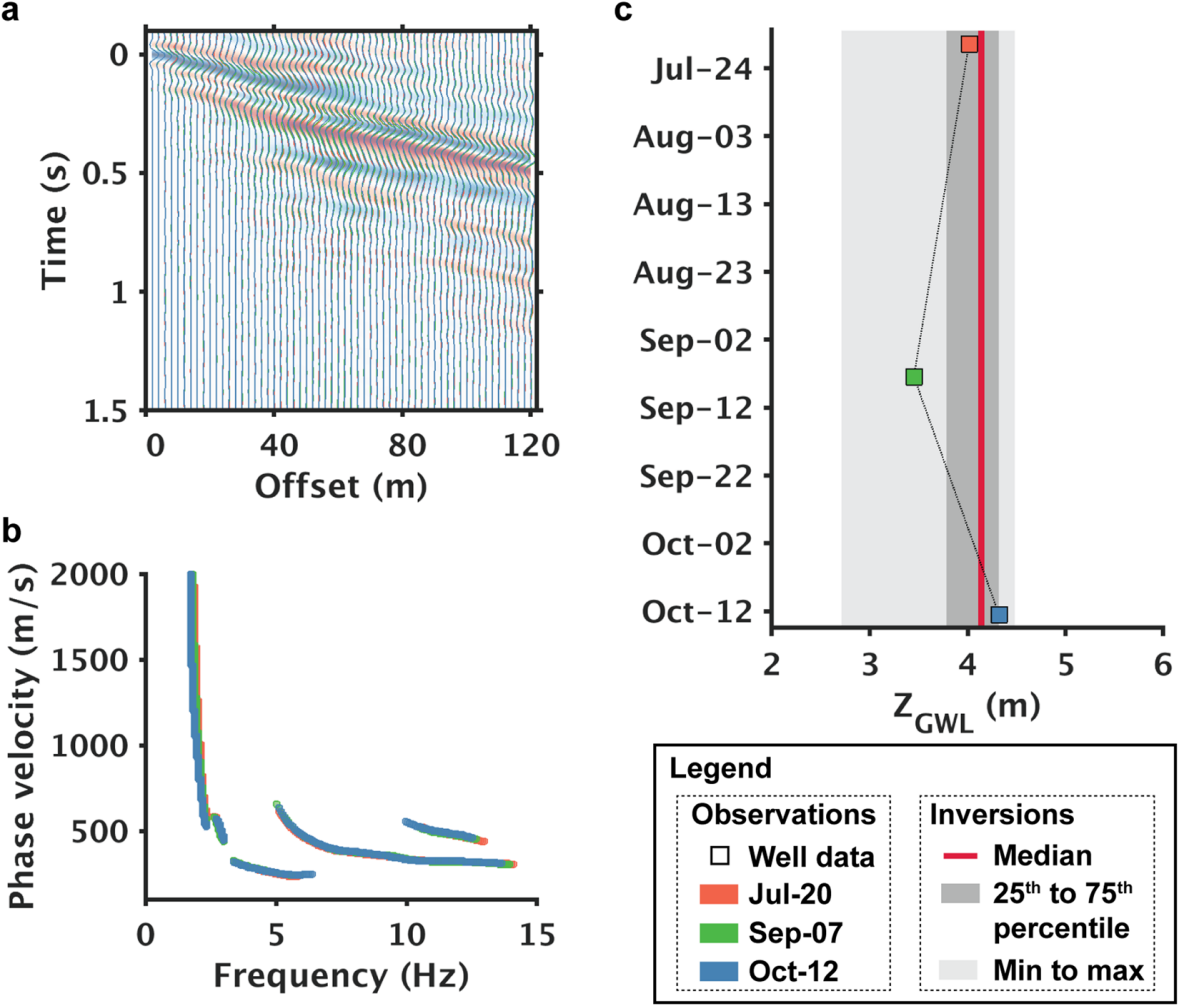
Time-lapse repeatability demonstration of ambient noise analysis in (**a**) space-time domain, (**b**) frequency-velocity domain, and (**c**) in terms of groundwater levels obtained from the model domain. Color sequence of red, green, and blue denote chronological orders of the monitoring period. In (**c**), the median, min-max range, and percentiles are calculated based upon all the topmost best-fitting models associated with the monitoring period.

Figure 4 demonstrates the repeatability of time-lapse imaging chiefly by comparing the groundwater levels retrieved from a monitoring well against what were obtained from the closest dark-fiber section. Note that no major rainfall events had occurred during the three-month monitoring period processed for this study, and as a result, the maximum changes in the measured water table did not exceed 0.9 m, on the order of our current error estimates for determination of surficial layer thickness (see Supplementary section, Table 1). The high repeatability of interferometric gathers, both in the offset-time domain (Fig. 4a) and frequency-velocity domain (Fig. 4b) suggests that such levels of variation will not be resolvable using this approach. The high time domain repeatability for single offsets are also shown in the Supplementary Materials (Fig. S4). An inspection in the model space (Fig. 4c) confirms the inversion’s insensitivity to these sub-meter changes. However, such repeatability provides assurance for reliably resolving larger changes in groundwater levels, because the well data, when examined over a year-long period, exhibit groundwater level changes up to 8 m. Such marked changes should be resolvable with the dark fiber array with a more extended monitoring period.

## Earthquake Seismology with a Dark Fiber DAS Array

Seismic network detection thresholds are highly heterogeneous, even across regions known for dense seismic monitoring like the western United States and Japan^50,51^, in part because broadband seismic stations are sited in hard-rock locations where background noise is low^52,53^. Areas of less-competent geology, like sedimentary basins, therefore correlate with poor catalog completeness; the magnitude of completeness is Mc 2–3 in the Sacramento and Southern San Joaquin Basins compared to Mc 0.5–2.4 in the San Francisco Bay Area or Mc 0.5–1.8 in Southern California^8,54,55^. Thus, despite the greater Sacramento area hosting significant gas production, underground gas storage, and high-volume waste water disposal, all of which can impact seismicity, the Sacramento Dark Fiber DAS array is located 30 km away from the nearest networked short-period seismometer (NDH) and 62 km away from the nearest broadband seismometer (AFD).

**Figure 5:**
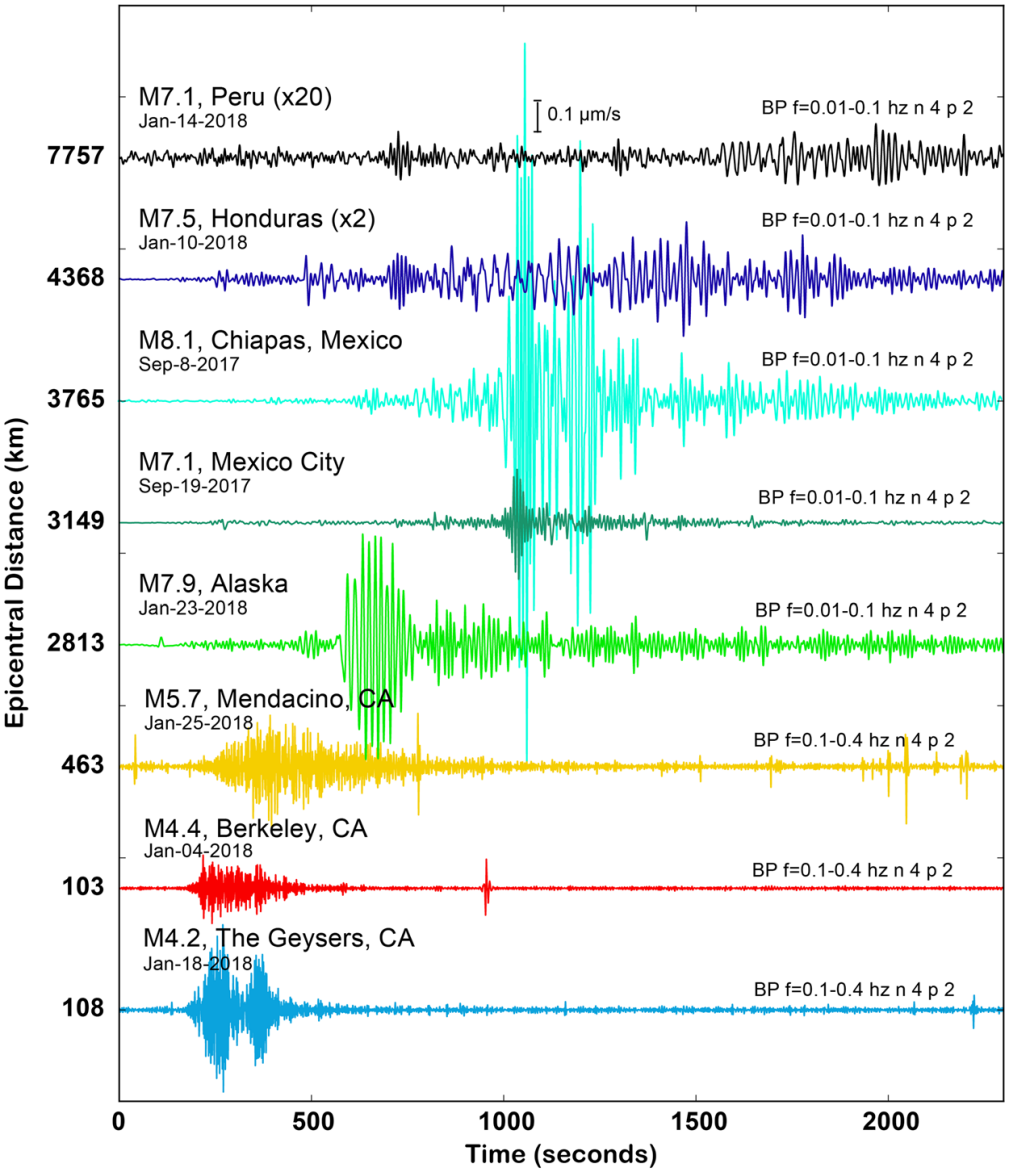
Example earthquakes recorded by the Sacramento Dark Fiber DAS array. The recorded data are plotted as strain-rate after multiplying by the gauge length (10 m) to convert to units proportional to velocity (1e-6 m/s), and have been averaged over 100 m of linear fiber length (50 traces) and then bandpass filtered in the 0.1–0.4 Hz range for regional events, and 0.01–0.1 Hz for teleseisms. Events are sorted by increasing epicentral distance from Sacramento. Earthquake amplitudes for the Peru and Honduras events are scaled by the factors in parentheses.

A relevant question when examining seismic events on telecommunications networks in contrast to fit-for-purpose installations is the impact of installation conditions. As recently demonstrated^16,17,19^, fiber installation in a standard plastic conduit does not preclude sufficient sensor coupling required for the detection of earthquakes, but the case of recording DAS data with repurposed telecommunications fiber is yet untested at regional scales. To explore this question with the Sacramento Dark Fiber DAS experiment we extract raw strain-rate waveforms for major global and regional earthquakes that occurred during the continuous recording interval (Fig. 5). We again use the linear quiet portion of the array shown in Fig. 1B and process the data by averaging 100 seismic traces (200 m section) and applying a bandpass filter to isolate the appropriate earthquake signals. To plot the raw strain-rate data in a more familiar unit we multiplied the data by a reference length equal to the gauge length (10 m) to convert to a unit that is proportional to velocity. We observed broadband DAS sensitivity to ground motion from earthquakes of varying magnitudes (M4.4–M8.1) and distances (100–7757 km). For example, in the case of the M7.5 Honduras event there is clear evidence of short period body waves and longer period surface waves over the two hour window following the origin.

While long period sensitivity is a major limitation of many inertial seismic sensors (e.g., accelerometers, short-period geophones, smartphone sensors), the long period response of DAS is currently a topic of active research with only limited available data^24^; teleseismic earth motion (strains near 1 × 10^−8^), for example, may be dominated by thermal expansion of the fiber-optic cable (strains on the order of 1 × 10^−6^) depending on the frequency studied as well as the depth, composition, and condition of the fiber-optic cable and conduit. Recent studies^24,56^ have used shallow hydrogeologic pump tests in a well with a fiber-optic cable to show that DAS has sensitivity to 9.4 × 10^−3^ Hz (period = 1080 seconds) oscillations in strain induced by the variable confining pressure, presumably due to Poisson effects. This subject is complicated by the known directionality of DAS cables^57,58^, which for the horizontal geometry of telecommunications dark fiber cables is theoretically insensitive to vertically-incident compressional motion (P-waves).

**Figure 6:**
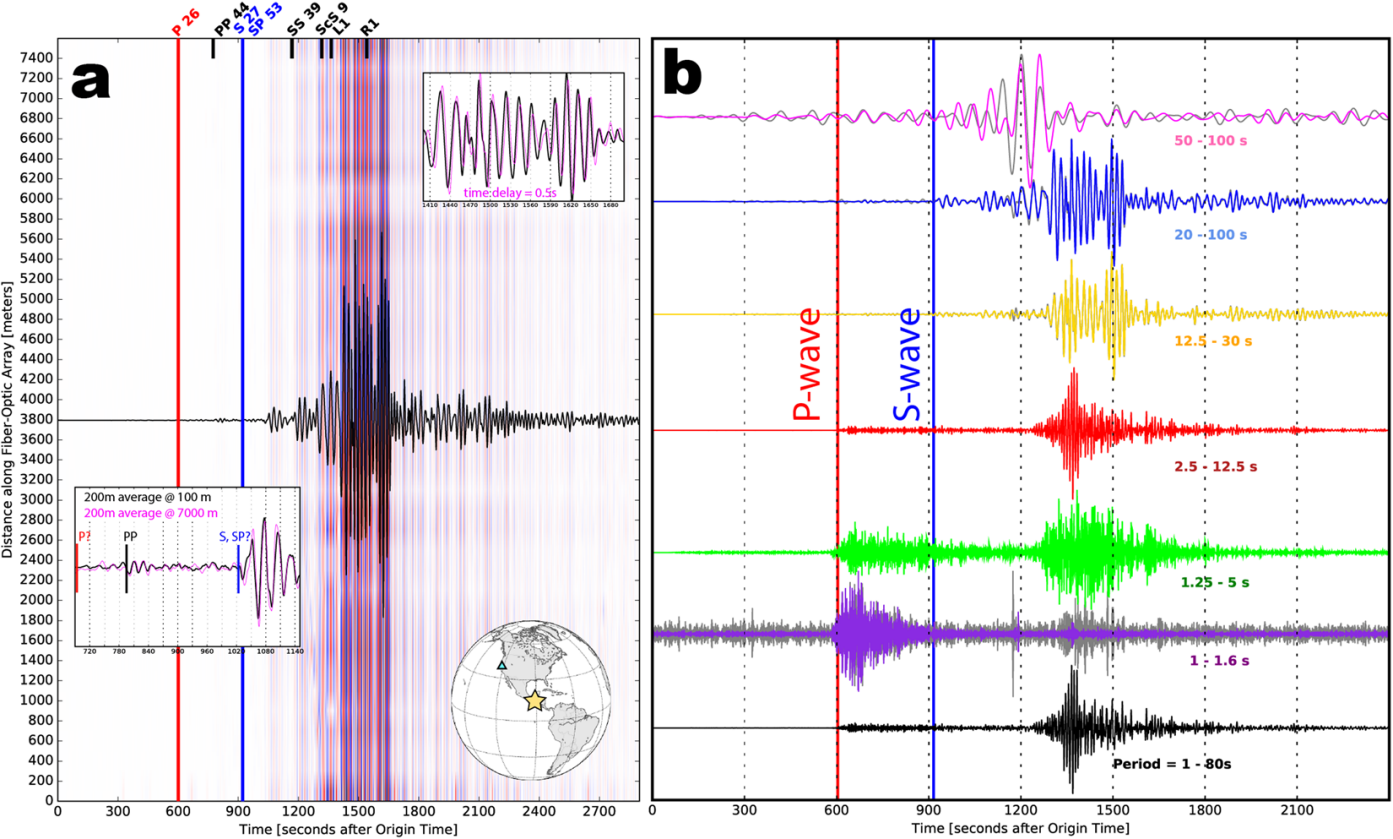
Teleseismic DAS recording of the M8.1 Chiapas, Mexico 2017-Sep-08 earthquake. (**a**) Seismic data for [black trace] one location and [red and blue] all locations from 0.0–7.6 km at a 2 m spacing (4001 traces total); top right inset shows surface waves arriving at the [black] south and [pink] north end locations of the array (backazimuth 120°), bottom left inset shows body waves arriving coincidently at both locations. A two-corner, zerophase, f = 0.01–0.5 Hz bandpass filter was applied. (**b**) Stacking 400 m or 200 consecutive DAS channels, color-coded by the bandpass filter applied to emphasize the broadband observation (1–100 seconds). Gray background traces show the single trace recording for cases that make a significant difference. Each of the traces is normalized to peak amplitude.

To explore the long period sensitivity of the Dark Fiber DAS array to teleseismic events, we extract raw strain-rate seismograms from the largest earthquake recorded during the experiment, the M8.1 2017-Sep-08 Chiapas, Mexico earthquake (Fig. 6). We observe broadband dispersive surface waves with strong energy at periods from 50–100 seconds. P-wave signal amplitude is lower than S-wave amplitude, perhaps because the sensor has minimal sensitivity to compressional particle motions for waves with incidence angles approaching 0° with respect to vertical (i.e. perpendicular to the fiber “broadside arrivals”). Nonetheless, the arrival times of major seismic phases are detected because of free surface scattering. Incidence angles of seismic phases are given in Fig. 6a. Differences in incidence angle also likely affect recorded amplitude, and appear to result in more coherent surface wave arrivals across the array (incidence angle = 0°) and as much as a 0.5 second delay time across the record section.

**Figure 7:**
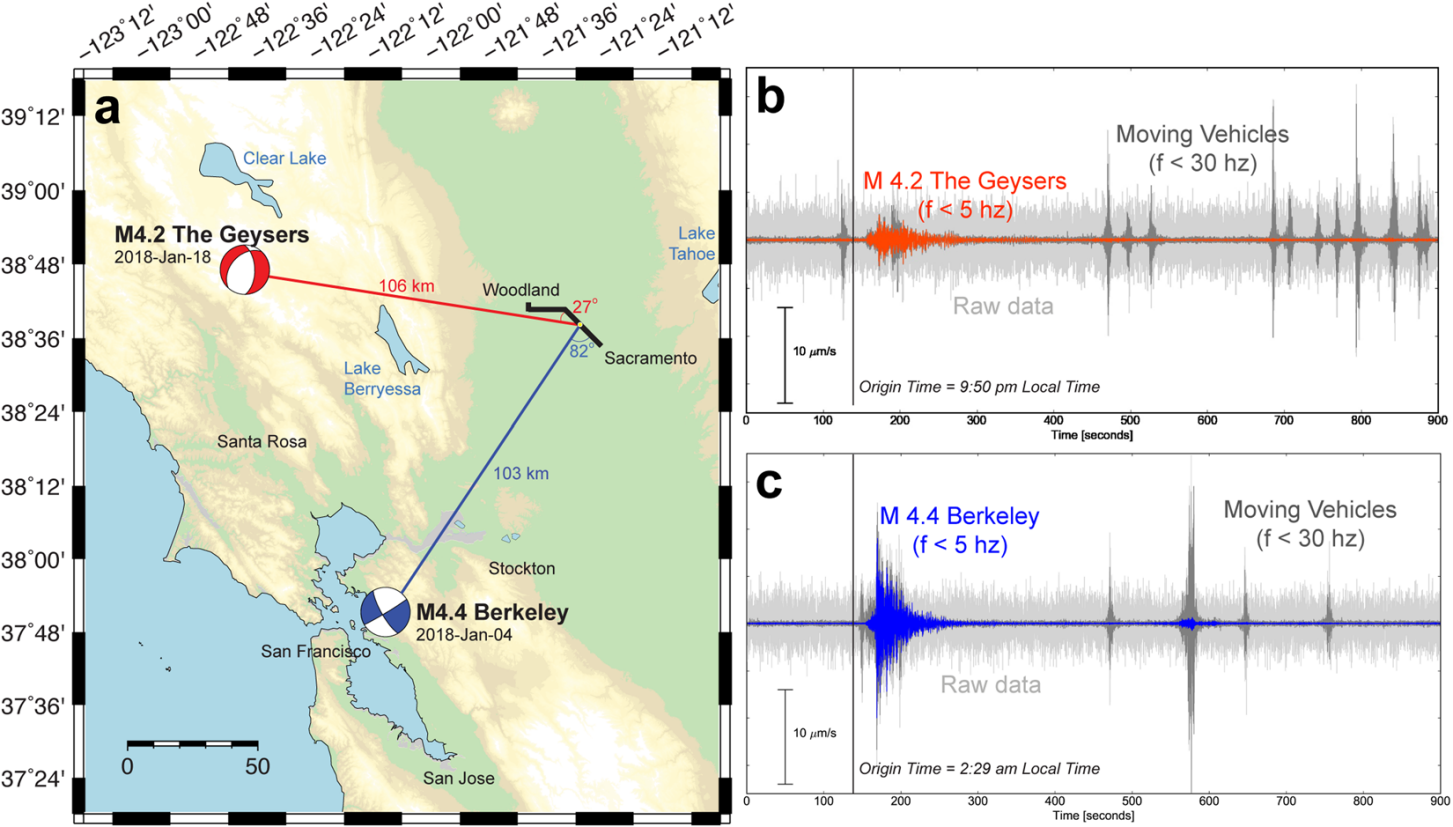
(**a**) Locations and focal mechanisms of the M4.2 2018-Jan-18 Geysers (red) and M4.4 2018-Jan-04 Berkeley (blue) earthquakes, which occurred approximately 100 km from the Sacramento Dark Fiber DAS array (black line). (**b**,**c**) Raw and lowpass filtered DAS strain-rate waveforms for these events averaged over 100 m (50 channels) at the yellow circle position shown in (**a**) (channel 4975 +/− 50 channels). Note the similarity between seismic and non-seismic signal amplitudes and the differences in frequency content.

Telecommunications cables are commonly routed along railways, roads, and through high noise urban areas. We find that major regional earthquakes (*M* ≈ 4) generate ground motions on the Sacramento array that have equal or lesser amplitude than local moving vehicles, however anthropogenic seismic signals typically are dominant in a higher frequency band (5–30 Hz). Figure 7 shows how the seismic signals from two different regional earthquakes (M4.22 Geysers 2018-Jan-18 and M4.38 Berkeley 2018-Jan-04) are easily discriminated from the local noise field based on spectral content. Higher frequencies of interest for local microearthquake analysis (f ≤ 50 Hz) will not always separate in this way.

The two earthquake records shown in Fig. 7 appear very different despite having been generated in similar sized ruptures and traveled similar distances to Sacramento. This may be due to source rupture depth differences (z = 2.4 km for The Geysers, z = 12 km for Berkeley), or the major differences in geologic structure along the raypath, but could also be the result of strong DAS axial sensitivity to energy in the direction of the fiber axis. We observe larger recorded amplitudes for shear waves from Berkeley than from The Geysers, but larger recorded amplitude for Rayleigh waves from The Geysers because of the more favorably oriented polarization.

**Figure 8:**
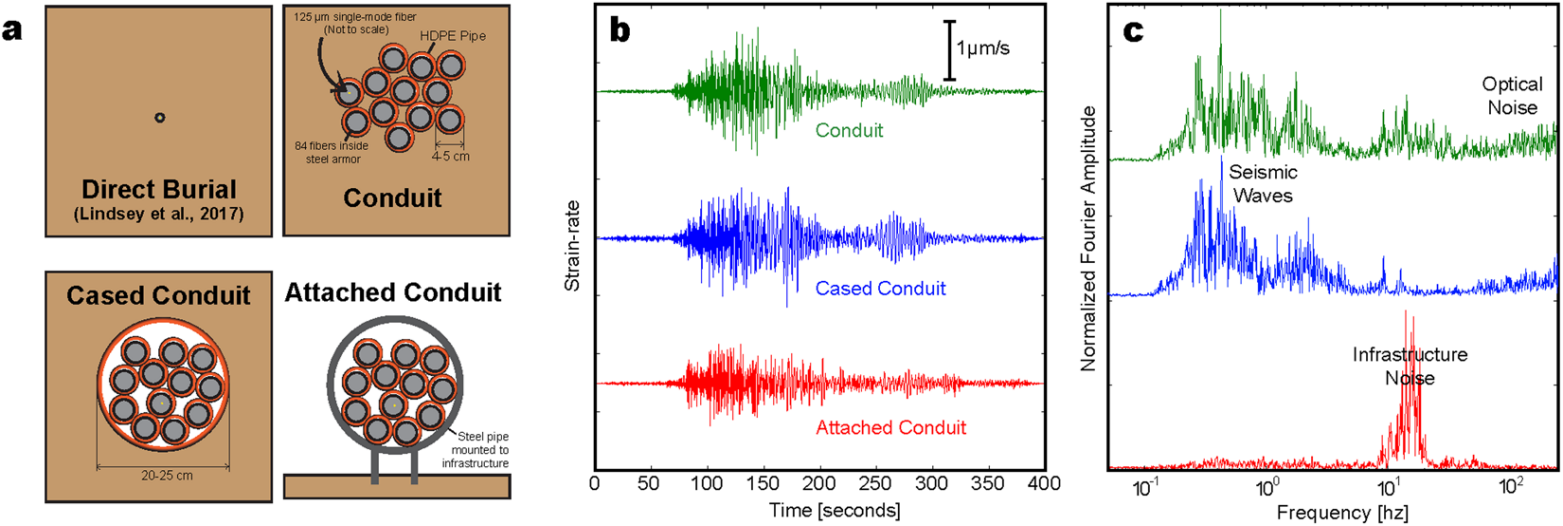
(**a**) Illustration of different installation geometries. (**b**) Earthquake (M4.2 Geysers 2018-Jan-18) trace comparison for each installation mode at Sacramento – trenched conduit (green), cased conduit (blue), attached conduit (red); strain-rate data are stacked over 100 m and filtered (BP 0.5–2 Hz n 4 p 2). (**c**) Normalized Fourier amplitude spectra for the waveforms shown in b.

We hypothesize that the method of installation (direct-burial, single conduit, conduit inside a larger conduit, conduit attached to infrastructure) has a significant effect on DAS recorded ground motion (see Fig. 8). The fiber-optic cable itself (gel-filled, aramid wrapped vs. loose-tube, polyethelene-jacketed vs. steel-armored, polyethelene vs. steel exterior) has each been shown to have only a small effect on recording quality at high frequencies^26^.

Installation information for the Sacramento Dark Fiber DAS array provides clues as to the heterogeneity of fiber-soil coupling across our experimental profile. Cable installation occurred in 1999–2000. Most of the fiber was pulled through one of 12 high-density polyethelene (HDPE) conduits (ID = 3.5–4 cm, wall thickness = 0.5 cm) that were buried together in a trench at 1–1.5 m and backfilled with soil before installing the fiber cable inside. Each fiber cable contains 84 gel-filled, loose-tube Corning LEAF fibers that are polythelene jacketed and steel-armored. The DAS data were recorded using a single 9/125 *μ*m single-mode fiber from one of these cables. In a few locations, trenching was not possible so directional boring was used to install a large casing conduit (ID = 20–25 cm, wall thickness = 0.4 cm), inside of which the 12 smaller conduits were pulled. Depth of boring varied between one meter and a few meters when navigating around various culverts, sections of road and railway, and other obstacles. In some instances the casing was not required, or a steel casing may have been used. A third mode of installation used for approximately 300 m of the dark fiber array involved attaching a 20–25 cm diameter steel casing directly to the elevated rail line where it crosses a section of protected wetlands, the Sacramento Bypass Wildlife Area. Inside this attached conduit, the 12 HDPE conduits were installed as the boring method described above.

Figure 8 shows DAS strain-rate earthquake waveforms (BP 0.5–2 Hz n 4 p 2) and normalized Fourier amplitude spectra for the M4.2 Geysers 2018-Jan-18 event stacked over 100 m of each of the three install modes. Any phase shifts between traces are due to these install locations being separated by as much as 7 km along the array. The conduit and cased conduit data show very similar seismic wave response to the ground motion centered in the f = 0.1–10 Hz range. Seismic signal amplitudes are observed to be on order with the optical noise at f ≥ 100 Hz. Data from attached section are noisier in a narrow frequency band centered on 12 Hz ± 3 Hz, perhaps caused by interaction of the incident seismic energy with the infrastructure and/or tube waves traveling in the attached conduit at air velocity. The trenched conduit shows a broader spectral response to near-surface scattering into surface waves, while the cased conduit is relatively insensitive to it. We should note that three installation conditions discussed in this study are certainly not a comprehensive survey. A large variety of techniques are used for fiber installation, ranging from direct cable burial to installation on utility poles; the impact on DAS recording for many have yet to be evaluated.

## Discussion

While the focus of our study was the specific utilization of installed telecom fiber probed by DAS for seismic sensing, our static imaging and monitoring results are consistent with and rely on broad advances in the field of ambient noise seismology applied to the near-surface. Beyond the foundational studies cited previously^38^, a variety of recent projects have utilized ambient noise approaches to probe hydrologic cycles^7,59–61^ and aquifer structure^62^ although typically using a sparse network of stations. Such studies have typically relied on the microseisms band as a noise source (0.1–1 Hz) and hence are observing averaged velocity perturbations over significant vertical extent, often to km depths. Despite this, such approaches have yielded convincing correlations with environmental parameters such as groundwater level^59^ although often for larger perturbations (10 s of m perturbations) in comparison to our study. In contrast, our experiment is densely sampled in space and utilizes infrastructure noise in the 0.5–18 Hz range; this enables adoption of surface wave inversion approaches utilized in the geotechnical community^46,47,63^ and the monitoring of very shallow features with some degree of depth resolution. Unfortunately, the minimal precipitation and small perturbations (1 m) in groundwater level which occurred during our study period precluded effective observation of hydrologic variations. Despite this fact, the static structure observed was consistent with our available ground truth dataset.

As mentioned previously, the ambient noise aspect of our study was also greatly enhanced by the broadband seismic signal generated by rail traffic co-linear to our measurement fiber. Recent seismic interferometry examples from the 2014 Belen experiment^64^ which utilized a more classical sensor array (4.5 Hz geophones) confirms the utility of rail noise as an imaging source for both P as well as S-wave imaging. These observations bode well for future dark fiber exploitation; since many telecom fiber installations use railway right-of-ways for installation, judicious selection of fiber paths can exploit this powerful source of seismic energy. Our experiment also provide an unusual case study of large (12k+ channel) array recording with dense receiver spacing (2 m). The only comparably sized experiments (5000+ sensors) are massive nodal deployments, which have recently been leveraged for similar event detection^34,65^ and ambient noise^66,67^ applications.

Outside of the validation datasets presented previously, prior studies in close proximity to our site are not extensive. Geotechnical evaluations, conducted in support of levee evaluation and construction broadly agree with our conclusions. At a location near our fiber profile, a past study evaluating levee safety in West Sacramento^68^ reported an average *V*_*s*30_ of 234 m/s which is broadly consistent with our surface wave inversion values of (210–280 m/s), a NEHRP class D soil environment.

As demonstrated in the prior sections, DAS-based seismic measurements acquired using dark fiber can provide a wealth of information relevant to near-surface seismic property estimation, hydrologic state, and natural seismicity. Measurements using dark fiber also have advantages in a host of situations ranging from marine to urban scenarios where classical seismic networks are challenging to execute.

An obvious strength of dark fiber DAS deployments, demonstrated in this study, is the potential to record data across long (10 s of km) transects at high spatial resolution without any required sensor installation or power source. Chains of such deployments could be utilized to provide true basin scale sensing; the Sacramento Basin’s central width (120 km) could be spanned using only 4 independent interrogation units and existing dark fiber resources, providing an unprecedented sensing resource. While basin scale hydrogeophysical monitoring studies using point sensors and ambient noise have been recently conducted^7^, the spatial resolution of such investigation is typically on the order of km due to sparse sensor distribution. Recent advances in large N processing approaches^32^ also offer strategies for leveraging dense arrays for detecting small seismic events.

A second advantage of utilizing dark fiber for seismic measurements is dense non-invasive coverage in urban areas where diverse deployment and permitting environments challenge classical acquisition strategies. The present study provides coverage spanning urban (Sacramento), suburban, and rural zones without the typical landowner permission effort, permitting, and survey work required for deployment in occupied areas.

While not demonstrated in this study, dark fiber can also be utilized for observations in the transition zone and offshore domains, areas where almost no measurements exist at present due to the high cost of tethered marine observatories including seismometers. Offshore DAS measurements would be particularly useful for improving the hypocenter accuracy for small events occurring on marine faults and earthquake early warning in subduction zones. Limitations of using DAS and dark fiber for offshore observations include (a) distance constraints for DAS measurements on single mode fiber for existing optical chains, currently in the range of 30–40 km, (b) the considerably higher cost for dedicated use of fibers in transoceanic cables, (c) the lower density of offshore cable routes which reduces coverage. Having said this, offshore dark fiber recording provides a clear future opportunity to extend the domain of seismological measurements into previously uninstrumented regions.

Despite these opportunities, challenges exist to fully exploit these conceptually novel sensing networks. Extremely large data volumes are among the most pressing, although solvable, problems; at maximum acquisition rates, a single interrogator can generate upwards of 20 TB/day. Combining these large N deployments with the long time periods required for ambient noise processing and monitoring yields raw data volumes that exceed the capacity of the computational infrastructure available to most researchers. The array in this study, which included 12000 channels sampled at 500 Hz, generated 128 TB of raw data in the first 3 months of operation and approximately 0.3 PB of raw data when the system was demobilized. Volumes of this size require careful consideration of data transport requirements, storage, archiving, and automated processing to be successfully utilized. Fortunately, on-going efforts to solve I/O and computational barriers in ambient noise studies^69,70^ provide a path to potentially handle the much larger datasets generated by dark fiber studies.

## Methods

### DAS system installation

The Silixa iDAS unit utilized in this study was installed on a vibration isolated table located in the West Sacramento PoP. Vibration isolation consisted of a 18 × 24′′ Nexus Breadboard (Thorlabs) with passive Sorbothane feet (Thorlabs) placed on a durometer 70 Sorbothane sheet. This assembly was placed on a rack shelf within the PoP cage where the utilized dark fiber was terminated in a standard fiber-optic patch panel with SC-UPC connection. Connection to the Silixa iDAS was made using an SC-UPC/SC-APC single-mode patch cable. An optical time-domain reflectometer (OTDR) was used to evaluate fiber integrity prior to recording. An OTDR trace measured a total loss of 20.8 dB over the full fiber length of 101 km at 1550 nm, or an average loss of 0.2059 dB/km.

### Details of data collection, computing, and processing infrastructure

As mentioned in the main manuscript, the data was collected at 500 Hz in the iDAS native format in the form of raw 1 minute records at 2 m spatial sampling. Files were written to a local USB3-connected 8 or 16 TB external hard drive. To maintain continuity of the dataset, hard drives were replaced on a weekly or biweekly basis and manually transferred from West Sacramento CA to Berkeley CA, where the data were uploaded to a local RAID storage server using the Globus protocol (https://www.globus.org/, last accessed: 2018-05-21) from a networked data transfer node. The storage server was linked to five RAID6 disk arrays and the full dataset was striped to improve performance. Primary processing was carried out on a 32-core GPU server connected to the storage server via a fast GB switch. Final results were visualized using a combination of MATLAB (Mathworks) and the ObsPy package.

### Processing Framework & Parameters for Ambient Noise Analysis

As mentioned in the primary manuscript, we adopted the ambient noise surface wave processing and inversion approach detailed in a prior DAS study^26^ with the steps shown in Supplemental Fig. S1. The overall workflow was initially derived from ambient noise analysis strategies developed over the last decade in the crustal imaging community^41^. Ambient noise interferometry was performed on sequential 120 m long subsections of the array to provide a combination of sufficient spectral resolution and a useful spatial resolution short enough for 1D analysis during inversion. In each subsection, the southernmost channel was treated as the virtual source and cross-correlated with the remainder of the channels.

Results shown in the ambient noise study are comprised exclusively of processing minutes when a train was passing near the selected array section; the train was the most energetic as well as broadband ambient noise source and selection of these records allowed for efficient processing. Train passes were identified for each section by scanning trace windowed RMS amplitude on the raw records. One minute records where the train was approaching or departing from a section were tagged for ambient noise processing. The train schedule was variable (2–6 passes per day) so each epoch utilized 40 minutes of train noise for analysis, independent of the number of days required to accumulate this stack. These N = 40 stacks were typically generated over approximately 10 days.

After selection and to prepare the raw records for noise correlation, static offsets and linear trends were removed, followed by temporal decimation down to a coarser sampling rate of 8 ms. Next, a temporal normalization with running-absolute-mean was applied over a 0.5-second running window. The frequency content of the data between 0.5 Hz and 18 Hz was then balanced with a spectral whitening step. Finally, in each of the 1-minute records, the noise of the virtual-source channel was cross-correlated with the rest of the channels’ records to form a common virtual-source gather. To achieve good SNR with a minimal stack count, phase-weighted stacking (*ν* = 0.5) was used and a stack count of 40 was sufficient for reaching temporal stability. Prior studies^71^ document the utility of phase weighted stacks in ambient noise analysis. A slant stack was then applied to the stacked common virtual-shot gathers to transform the data from space-time domain to frequency-velocity (dispersion) domain.

The input to all inversions were experimental multimodal dispersion curves extracted from the frequency-velocity domain images. Given the very large number of datasets generated including 100 s of sub-arrays over 10 s of epochs, automation, rather than hand-picking, was a necessity. A simple algorithm was developed to pick the dispersion curves by 2D scans for local maximums with a lower threshold. As can be seen from the example in Fig. 2c, the presence of strong modes besides the fundamental forced adoption of a multimodal inversion approach. One weakness of the auto-picking approach is handling of the low frequency ends of the dispersion curves; in these cases, the auto-picks often are biased to high velocities which can distort (increase) the velocity of the bounding 1/2 space.

As discussed in prior work^26^, mode-labeling can present a challenge for this class of DAS dataset hence we adopted an inversion approach which did not require explicit mode numbering. For our objective function in the inversion, we utilized a recently developed formulation developed by Maraschini and collaborators^46,47^. Their novel approach, which we refer to as the Haskell-Thomson determinant method, searches for models that can minimize the determinant of a model-predicted propagator matrix whose frequency and velocity terms are replaced with the experimental dispersion curves. We refer the interested reader to prior descriptions of the algorithm^46,47^ and a prior example of applying it to DAS data^26^. The advantage of this approach is that (a) the objective function can be very efficiently evaluated without root finding, thus allowing global search, and (b) non-labeled multimodal dispersion data can be utilized as an input.

Because of the nonlinear nature of the problem and the low computational costs of the Haskell-Thomson determinant method, Monte Carlo sampling was used^46^ as part of search and model selection. We adopted a sparse parameterization for the search problem assuming a four layer model for each array subsection and solved for *V*_*s*_ and layer thickness for each location. For each 1D inversion, a Monte Carlo pool size of 1 × 10^6^ models was used. Search bounds of the model parameters are shown in Table 1. Each model parameter was assumed to be uniformly distributed in the sampling process within the bounds, with the restriction that all models were required to have increasing *V*_*s*_ with depth, likely a valid assumption in this geology. As can be seen in Table 1 and Fig. 2d, the bounds are not tight and allow effective model exploration. All models were ranked by L1 misfit; the optimum models were selected for interpretation. Each set of the inversions took 1.5 minutes on 24 cores (2.3 GHz Intel Xeon processors).

**Table 1:** Upper and lower bounds in Monte Carlo sampling of the inversion variables.

Layer	${{\boldsymbol{V}}}_{{{\boldsymbol{s}}}_{{\boldsymbol{\min }}}}$ (m/s)	${{\boldsymbol{V}}}_{{{\boldsymbol{s}}}_{{\boldsymbol{\max }}}}$ (m/s)	*h*_*min*_ (m)	*h*_*max*_ (m)
1	100	300	1	6
2	200	800	2	30
3	500	2000	2	30
4	1500	2500	—	—

### Processing Framework & Parameters for Earthquake Analysis

We identified earthquake records in the continuous raw DAS dataset using catalogued origin times and approximate travel times to Sacramento for the 1-D iasp91 Earth velocity model. 1-minute duration earthquake records were merged together, and then, if desired, stacked with a mean average of traces over a specified length (100 m = 50 traces averaged and plotted at the midpoint), prior to the application of a specified bandpass filter to remove unwanted or uninteresting signals. In Fig. 5a, the iasp91 model was again used to calculate the phase arrival angles with respect to vertical. Figure 5b shows one stacking effect.

### Data on Reference Wells

Data on the reference groundwater monitoring well discussed (W1) was acquired from the CASGEM database. As mentioned in the text, the well is referred to as the Sac Bypass Shallow Well (State well ID 09N04E20-N001M and CASGEM ID 25619). The well is located in Yolo County at 38.6062 N, −121.5602 W with a surface elevation of 6.55 m (21.49 ft) above sea level; the well is associated with the Yolo County Water Resources Association (WRA) with measurements conducted by the CA Department of Water Resources (DWR). The well is completed with slotted PVC and a sandpack over a 6.1 m (20 ft) interval from 24.4 to 30.5 m below ground surface. Water table depths were measured manually at an irregular intervals (every 1 to 3 months) with a reported accuracy of 0.3 cm (0.01 ft).

## Electronic supplementary material

Supplementary Section

## Data Availability

Due to the very large size of this dataset, only decimated raw components or processed subsections are available upon request.
