# Supplementary material for: Distributed Acoustic Sensing Using Dark Fiber for Near-Surface Characterization and Broadband Seismic Event Detection
Source: Sci Rep. 2019 Feb 4;9:1328. doi: 10.1038/s41598-018-36675-8 (PMC6362068; doi:10.1038/s41598-018-36675-8)
Supplement: Supplementary file 1 — Supplementary Section [file 41598_2018_36675_MOESM1_ESM.pdf]

Supplementary Information for: Distributed Acoustic Sensing Using Dark Fiber for Near-Surface Characterization and Broadband Seismic Event Detection

Jonathan B. Ajo-Franklin[1,*,+], Shan Dou[1,+], Nathaniel J. Lindsey[1,2,+], Inder Monga[3], Chris Tracy[3], Michelle Robertson[1], Veronica Rodriguez Tribaldos[1], Craig Ulrich[1], Barry Freifeld[1], Tom Daley[1], and X.S. Li[4]

[1]Lawrence Berkeley National Laboratory, Energy Geoscience Division
[2]University of California, Berkeley, Earth and Planetary Sciences Department
[3]Lawrence Berkeley National Laboratory, Energy Sciences Network
[4]Lawrence Berkeley National Laboratory, Computational Research Division
*jbajo-franklin@lbl.gov
+these authors contributed equally to this work

November 20, 2018

Ambient Noise Analysis Processing Flow

Fig. S1 below provides a schematic of the ambient noise processing steps described in the methods section. All relevant parameters for the processing steps are available in the prior section.

S-Wave Model Fit Evaluation

A challenge when applying inversion algorithms automatically to a large number of data subsets is appraisal of solution quality. In the case described in this study, the large data volume, including hundreds of sub-arrays imaged over many temporal epochs, precludes manual inspection of all results. For the static pseudo 2D inversion profile in Fig. 3, we inspected all results for quality (e.g. fit on the fundamental mode). Fig. S2 shows solution evaluation for four locations spanning the 2D profile with the top row (Fig. S2A) showing the experimental dispersion curve autopicks (black) superimposed on the modal solutions predicted by the best fitting V_s model (red symbols). The lower row (Fig. S2B) shows the best fit solution (red line) in the context of the top 0.1% of the MC solution pool colored by relative misfit and the inversion bounds (dashed yellow line).

As can be seen in Fig. S2A, the fundamental mode and a range of higher order modes are visible at all locations. A variety of ”mode kissing” scenarios are present, where the fundamental crosses or merges with the first or higher order overtones; these situations present a challenge for both auto pickers and the inversion algorithm. Despite this, the auto picker generally captures the fundamental and higher frequency components of the low order modes well. The experimental dispersion curves extracted from the fundamental generally match the modal curves predicted from the inverted (best fit) V_s models; we should note that the Thomson-Haskell determinant objective function used to select the best fit model and rank MC solutions is independent of the modal solver used to predict the dispersion curves in Fig. S2A. The auto picker has more difficulty with weak higher order (≥ 2) modes and hence they are not utilized in the inversion. We should also note, as mentioned in the main manuscript, that the auto picker tends to over-estimate velocities in the low frequency regime, thus biasing the bounding lower 1/2 space to higher velocity values.

S-Wave Model Uncertainty Analysis

The uncertainty associated with the V_s models derived from surface-wave inversion can be estimated by analyzing the family of models which come close to fitting the observed data. As mentioned in the Methods section, our MC search assumes a uniform prior distribution of model parameters. We analyze the statistics of the top 0.1% of models by misfit (”accepted models”) to evaluate the certainty of estimated model parameters. While this analysis is not as rigorous as a full posteriori model covariance analysis, it does provide some intuition as to the uncertainty levels for different model components. We should also note that the analysis which follows captures uncertainty related to multiple models which fit the picked dispersion curves; we do not have a good way at present of capturing the correlated error related to bias in the dispersion curve picking process and hence the uncertainty estimates should be viewed as a lower bound.

This analysis is illustrated in Fig. S3 for the 120 m sub-array starting at channel 2900 (i.e 800 m from the southern end of the red profile shown in Fig. 1B). In panel A, the distribution of V_s and base depth for each of the model layers is shown for the top 0.1% best-fit models from the original 1×10^6 models in the MC pool. This distribution indicates that both V_s and layer base are best resolved for the top 30 m of the profile. In panel B, the median of the model ensemble is adopted as the reference value for investigating repeatability. The 25th and 75th percentiles are used as upper and lower bounds. Hence, deviations from the reference are treated as model space uncertainty. Associated values are detailed in Table 1. As mentioned, better constrained parameters are obtained for the top section of the profile. In layer 1, whose base is consistent with the position of the water table and is interpreted to be the unsaturated zone, model variations in both V_s and interface depth are on the order of 17%, or approximately a ± 0.8 m uncertainty in groundwater level estimation.

The second interface could be associated with the transition from either fine sand to gravel sand or gravel sand to brown clay. More ground truth information such as logs of petrophysical properties would be required to confirm this interpretation. Model variability in V_s for this layer, however, is only of 3-3.8%, which shows that velocity changes greater than approximately 4% could be resolved. The remaining interfaces of the model most likely represent the boundary between the brown and the blue clays described in the geotechnical log shown to the right of panel B. As can be seen, uncertainties as to the interface depth and properties of the deeper horizons ($z \geq 30m$) are considerably greater than for the shallower horizons (layers 1 & 2), as has been seen for prior studies with similar noise sources[1] and geometries. As mentioned previously, the high velocity for the deepest layer (bounding 1/2 space) is likely due to low-frequency bias in the dispersion curve auto picking process; unfortunately this bias is not captured in figure S3 which considers only model-domain uncertainty.

Ambient Noise Waveform Repeatability

Fig. S3 shows the high repeatability observed in time-domain waveforms over the course of the experiment for 9 temporal epochs from July 24th to Oct. 12th. The data is extracted from cable channels 2700-2750, approximately 400 m from the south end of the red profile shown in Fig. 1B with an assumed virtual source to the south. Each trace corresponds to a stack of 40

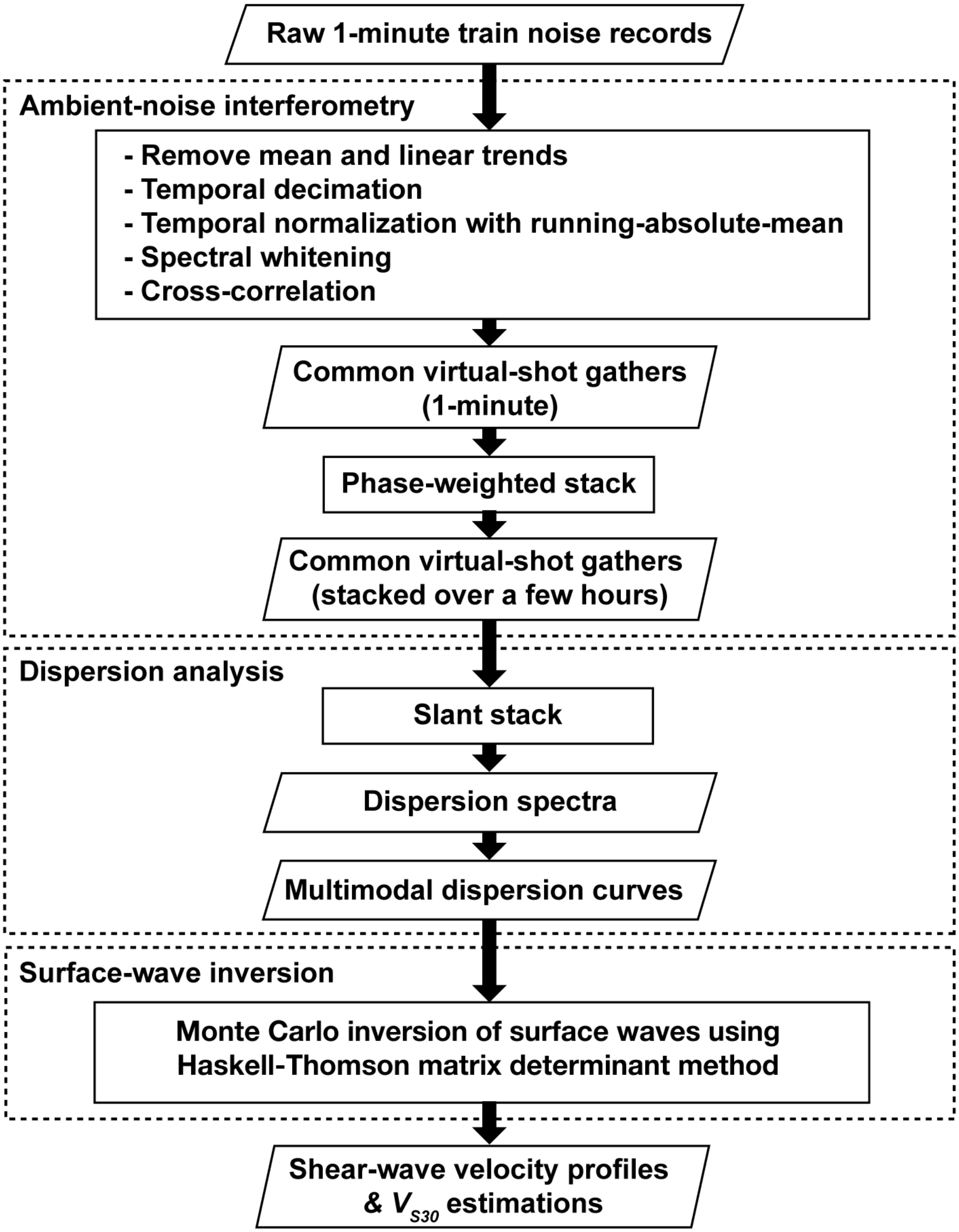

Figure S1. Ambient noise processing flow for near-surface V_s reconstructions.

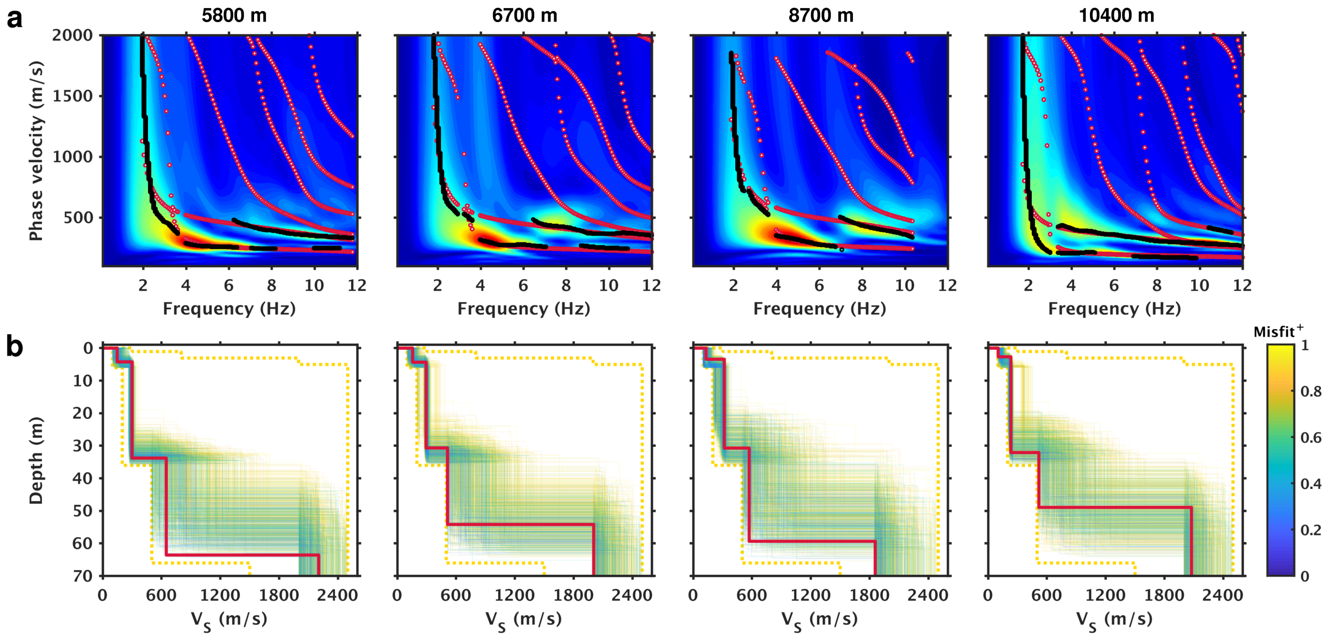

Figure S2. Examples of experimental dispersion observations, inverted S-wave models, and misfit with predicted dispersion curves: (A) Experimental dispersion data in the background image for four locations along the profile, 120 m subarrays located 5800 m, 6700 m, 8700 m, and 10,400 m from the interrogator. The superimposed black symbols are the automatically picked multimodal dispersion curves. The red symbols are the dispersion curves predicted from the best-fit V_s model. (B) Inverted V_s models for the location indicated with the solid red line showing the best-fit model, the light colored curves showing the top 0.1% of models from the MC pool colored by relative misfit, and the dashed yellow lines showing inversion constraints.

Table 1. Error estimates of top 0.1% best-fit models.

	V_s (m/s)			Base depth (m)		
**Layer**	**Median**	**25th percentile-median**	**75th percentile-median**	**Median**	**25th percentile-median**	**75th percentile-median**
1	147.7	-26.3 (-17.8%)	27.4 (18.55%)	4.62	-0.82 (17.75%)	0.81 (17.53%)
2	283.6	-8.5 (-2.99%)	10.9 (3.84%)	32.24	-2.03 (6.30%)	1.2 (3.72%)
3	790.9	-152.9 (-19.33%)	207.4 (26.22%)	52.88	-4.8 (9.07%)	4.37 (8.26%)
4	2167.1	-96.5 (-4.45%)	112.7 (5.2%)			

train passes (1 minute of data each) and incorporates the same processing flow as utilized in the primary manuscript. The left side shows the repeatability results for a 50 m offset from the virtual source while the right side shows a similar result for a 120 m offset.

Fig. S4B shows all corresponding epochs superimposed and plotted in different colors. As can be seen, the stacked waveforms are highly stable with no qualitative variability, except perhaps small phase variations deeper in the coda.

Secondary Data Sources

Historical groundwater level data was obtained from public domain sources including the California Statewide Groundwater Elevation Monitoring (CASGEM) database [http://www.water.ca.gov/groundwater/casgem/]. Real-time groundwater data was obtained from the California Data Exchange Center (CDEC) [https://cdec.water.ca.gov/]. Completion report and driller's logs were obtained from the CA Department of Water Resources (DWR) Well Completion Report Map Application [https://gis.water.ca.gov/app/wcr/]. Topographic data and map layers used in Fig. 1 were obtained from the USGS National Map project [https://nationalmap.gov/].

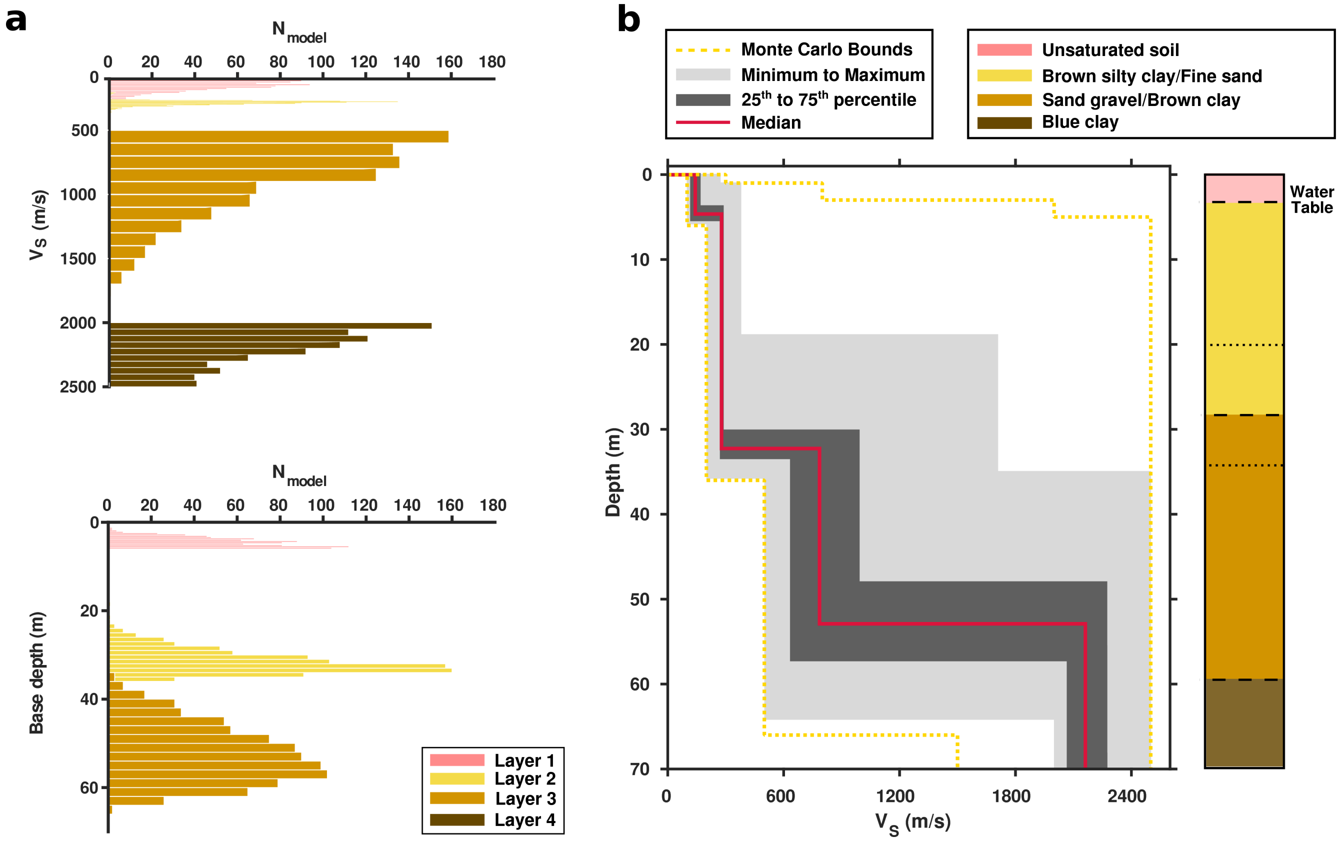

Figure S3. V_s Model Uncertainty Analysis: Panel (A) shows parameter value histograms for the top 0.1% of models (by L1 misfit) selected during MC search, for V_s estimation (top) and interface depth (bottom). Note that histogram bin sizes differ for parameters. Panel (B) shows the median V_s model (red) as well as the $25^{th}/75^{th}$ percentile bounds (dark grey), min/max bounds (light grey) and search bounds (dashed yellow). To the far right of panel (B) is the interpreted lithologic column from well W2. All results are shown for a 120 m sub-array starting at line channel 2900, 800 m north of the southern end of the red profile shown in Figure 1B.

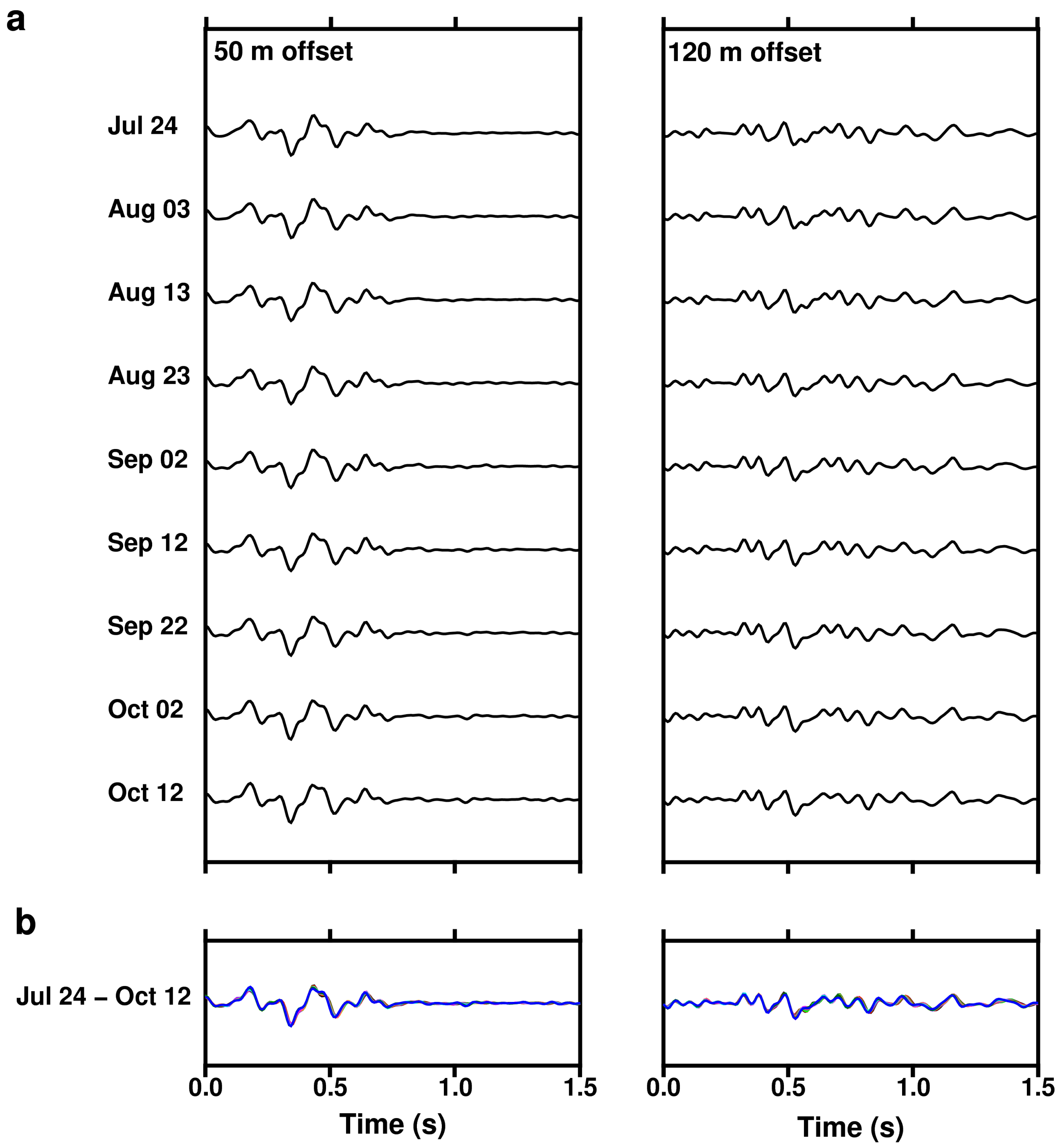

Figure S4. Variability in waveforms recovered from ambient noise processing: (A) Time-domain waveforms for 9 temporal epochs for two virtual source offsets (50 and 120 m); (B) Waveforms overlaid.

References

1. Dou, S. *et al.* Distributed acoustic sensing for seismic monitoring of the near surface: A traffic-noise interferometry case study. *Scientific reports* **7**, 11620 (2017).
